# Myocardial strain in healthy adults across a broad age range as revealed by cardiac magnetic resonance imaging at 1.5 and 3.0T: Associations of myocardial strain with myocardial region, age, and sex

**DOI:** 10.1002/jmri.25280

**Published:** 2016-04-22

**Authors:** Kenneth Mangion, Guillaume Clerfond, Christie McComb, David Carrick, Samuli M. Rauhalammi, John McClure, David S. Corcoran, Rosemary Woodward, Vanessa Orchard, Aleksandra Radjenovic, Xiaodong Zhong, Colin Berry

**Affiliations:** ^1^ BHF Glasgow Cardiovascular Research CentreUniversity of GlasgowUK; ^2^ West of Scotland Heart and Lung CentreGolden Jubilee National HospitalClydebankUK; ^3^ Clinical PhysicsNHS Greater Glasgow and ClydeGlasgowUK; ^4^ MR R&D CollaborationsSiemens HealthcareAtlantaGeorgiaUSA

**Keywords:** healthy volunteers, myocardial strain, displacement encoding with stimulated echoes

## Abstract

**Purpose:**

To assess myocardial strain using cine displacement encoding with stimulated echoes (DENSE) using 1.5T and 3.0T MRI in healthy adults.

**Materials and Methods:**

Healthy adults without any history of cardiovascular disease underwent magnetic resonance imaging (MRI) at 1.5T and 3.0T within 2 days. The MRI protocol included balanced steady‐state free‐precession (b‐SSFP), 2D cine‐echo planar imaging (EPI)‐DENSE, and late gadolinium enhancement in subjects >45 years. Acquisitions were divided into six segments; global and segmental peak longitudinal and circumferential strain were derived and analyzed by field strength, age, and gender.

**Results:**

In all, 89 volunteers (mean age 44.8 ± 18.0 years, range: 18–87 years) underwent MRI at 1.5T, and 88 of these subjects underwent MRI at 3.0T (1.4 ± 1.4 days between the scans). Compared with 3.0T, the magnitudes of global circumferential (–19.5 ± 2.6% vs. –18.47 ± 2.6%; *P* = 0.001) and longitudinal (–12.47 ± 3.2% vs. –10.53 ± 3.1%; *P* = 0.004) strain were greater at 1.5T. At 1.5T, longitudinal strain was greater in females than in males: –10.17 ± 3.4% vs. –13.67 ± 2.4%; *P* = 0.001. Similar observations occurred for circumferential strain at 1.5T (–18.72 ± 2.2% vs. –20.10 ± 2.7%; *P* = 0.014) and at 3.0T (–17.92 ± 1.8% vs. –19.1 ± 3.1%; *P* = 0.047). At 1.5T, longitudinal and circumferential strain were not associated with age after accounting for sex (longitudinal strain *P* = 0.178, circumferential strain *P* = 0.733). At 3.0T, longitudinal and circumferential strain were associated with age (*P* < 0.05). Longitudinal strain values were greater in the apico‐septal, basal‐lateral, and mid‐lateral segments and circumferential strain in the inferior, infero‐lateral, and antero‐lateral LV segments.

**Conclusion:**

Myocardial strain parameters as revealed by cine‐DENSE at different MRI field strengths were associated with myocardial region, age, and sex. J. Magn. Reson. Imaging 2016;44:1197–1205.

Displacement encoding with stimulated echoes (DENSE) is a magnetic resonance imaging (MRI) method that directly quantifies left ventricular (LV) mechanics within myocardial regions with high spatial and temporal resolution.^1^ LV ejection fraction (LVEF) and wall motion score reflect displacement of LV borders, provide global measures of heart pump function, and are the standard imaging methods for describing LV function.^2^ Global longitudinal strain (GLS) has independent prognostic significance compared with LVEF in myocardial infarction survivors,^3^, ^4^ and in patients with cardiomyopathy.^5^, ^6^ Echocardiography is the standard‐of‐care for imaging LVEF and GLS. However, compared with echocardiography, MRI of LV systolic function is less dependent on operator technique and patient habitus (eg, acoustic windows) and has increased precision.^7^ MRI also enables characterization of myocardial pathology and therefore enables direct regional measurement of contractility that can be spatially matched with pathology. However, the variation in strain values within a healthy population with age and field strength has still to be established.

There are a number of ways of assessing myocardial strain with MR, including myocardial tagging,^8^ strain‐encoding imaging (SENC),^9^ phase contrast (PC) imaging,^10^ and DENSE.^1^ As a relatively new MR technique in this family, cine DENSE is capable of revealing pixel‐by‐pixel displacement and subsequently strain information of myocardial tissue from the phase data at each cardiac phase.^1^, ^11^, ^12^, ^13^ Our aim was to assess the relationship between myocardial strain as revealed by DENSE with age and sex, in a reasonably large group of healthy volunteers at two field strengths. We hypothesized that magnitudes of strain are associated with age and sex, independent of field strength.

## Materials and Methods

### Study Population

The study was approved by the regional ethics committee. Healthy volunteers aged at least 18 years with no prior medical history (including cardiovascular health problems, medication, or systemic illness) were invited to participate by placing advertisements in public buildings (eg, hospital, university). The other exclusion criteria included standard contraindications to MR (eg, metallic implants and metallic foreign body) and known or suspected pregnancy. Written informed consent was subsequently obtained from prospective participants. A 12‐lead electrocardiogram (ECG) was obtained in all subjects and a normal ECG was an inclusion criterion. Patient characteristics were recorded and body surface area was calculated with the Dubois formula.

### MR Acquisition

Participants underwent MRI at 1.5T (MAGNETOM Avanto, Siemens Healthcare, Erlangen, Germany) located in a hospital Radiology Department and at 3.0T (MAGNETOM Verio, Siemens Healthcare) in a university research center. At both field strengths, images were acquired using an anterior phased‐array body coil (12‐element and 16‐element at 1.5T and 3.0T, respectively) and a posterior phased‐array spine coil (24‐element).

### MR Protocol

LV dimensions were assessed using balanced steady‐state free‐precession (b‐SSFP) cinematographic breath‐hold sequences. Typical imaging parameters are shown in Table 1. The heart was imaged in multiple parallel short‐axis planes 8‐mm thick separated by 2‐mm gaps, as well as in the 2‐chamber, 3‐chamber, and 4‐chamber long‐axis views.

**Table 1:** Typical Imaging Parameters, at 1.5T and 3.0T MR Field Strengths

b‐SSFP	1.5T	3.0T
TR (msec)	3.3	3.4
TE (msec)	1.2	1.5
FoV (mm)	340	340
Flip angle (degree)	80	50
Slice thickness (mm)	7	7
Resolution (mm)	180×256	256×256
Bandwidth (Hz/pixel)	930	977
**DENSE**	**1.5T**	**3.0T**
TR (msec)	32.5	27.34
TE (msec)	7.97	6.63
FoV (mm)	360	360
Voxel size (mm)	3.2×3.2×8	3.2×3.2×8
Flip angle (degree)	20	20
Bandwidth (Hz/pixel)	1207	1207
Displacement encoding Frequency (π/mm)	0.2	0.2
Triggers per breath‐hold	8	8
EPI factor	8	8
Segments per cardiac frame	16	16
Shimming method	Automatic	Manual

TR: repetition time (msec); TE: echo time (msec); FoV: field of view (mm).

A 2D echo planar imaging (EPI) DENSE sequence (work‐in‐progress sequence 611, Siemens Healthcare) was used to acquire mid‐ventricular short‐axis and 4‐chamber long‐axis views. Typical imaging parameters are shown in Table 1. Through‐plane dephasing and 2‐point complementary spatial modulation of magnetization (CSPAMM) were used for artifact suppression during DENSE acquisition.^14^ Fat suppression was carried out using a fast water excitation option provided by the vendor. The readout and phase‐encoding direction of displacement were acquired in a single breath‐hold.

Participants over 45 years of age had their renal function checked and if the estimated glomerular filtration rate (eGFR) was >30 mls/min/1.73 m^2^ gadolinium contrast was administered (0.15 mmol/kg per bolus of gadolinium diethyltriaminepentaacetic acid [Gd‐DTPA], Magnevist, Bayer Healthcare, Berlin, Germany). Late gadolinium enhancement images covering the entire LV were acquired 10–15 minutes after intravenous contrast agent administration using segmented phase‐sensitive inversion recovery (PSIR) turbo fast low‐angle shot sequence.

### Image Analysis

Datasets were anonymized to ensure operators were blinded to all other data. Project data were coordinated by S.R. The absence of late gadolinium enhancement (myocardial fibrosis or scar) was determined qualitatively by visual assessment by D.C. (>3 years MRI experience) and C.B. (>10 years MRI experience). The absence of myocardial late gadolinium enhancement was another requirement for inclusion of the data in this analysis.

LV mass and function were analyzed in randomly ordered, deidentified scans by three MRI‐trained cardiologists (MRI experience: G.C. 5 years, K.M. 3 years, D.S.C. 3 years) using computer‐assisted planimetry (Syngo MR, Siemens Healthcare).

The LV was segmented using the anterior right ventricular‐LV insertion point as the reference point. The mid‐left ventricular short axis (six segments: anterior, antero‐septal, infero‐septal, inferior, infero‐lateral, and antero‐lateral) and horizontal long axis (6 segments: basal infero‐septal, mid‐infero‐septal, apical septal, apical lateral, mid‐antero‐lateral, and basal‐antero‐lateral) DENSE data were analyzed using the CIM_DENSE2D software (University of Auckland, New Zealand),^15^ which provided longitudinal and circumferential strain. The underlying principles of the analysis included segmentation, phase unwrapping, displacement extraction, and strain calculation. Global and segmental myocardial circumferential (E_cc_) and longitudinal (E_ll_) strain were analyzed by three trained observers (K.M., G.C., C.M.) in a random order. Image quality and artifact scoring were carried out by two operators (K.M., G.C.) and when there was discordance, X.Z. acted as a blinded independent adjudicator.

### Statistical Analysis

Statistical analysis was performed using SPSS software (Chicago, IL, v. 22). Normality was tested using the Shapiro‐Wilk test. Continuous variables are expressed as mean ± standard deviation (SD). For comparison of two or more normally distributed variables, Student's *t*‐test and analysis of variance (ANOVA) with Tukey post‐hoc analysis were used. The population was divided per tertile of age to compare strain between groups. Interobserver reproducibility was assessed using Bland–Altman statistics. *P* < 0.05 was considered statistically significant.

## RESULTS

### Characteristics of the Study Participants

A total of 90 subjects underwent cardiac MRI. One male had an incidental finding of high *T*_1_ in the antero‐septal LV wall in the territory of the left anterior descending coronary artery. A clinical assessment disclosed a history of exertional chest pain suggestive of angina. The elevated *T*_1_ may have reflected myocardial edema secondary to ischemia. This patient was excluded from the analysis and referred for clinical investigation.

**Figure 1:**
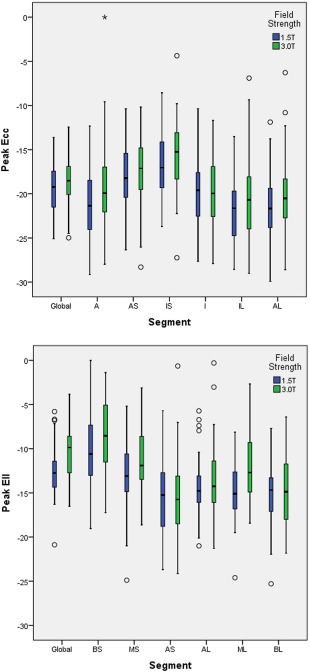
Global and segmental circumferential **(a)** and longitudinal **(b)** strain values at different field strengths. The circles in the figures represent participants outwith the confidence intervals.

The characteristics of the remaining participating subjects (*n* = 89, including nine individuals ≥70 years) and their LV mass and function are presented in Tables 2 and 3, respectively. Global and segmental strain values are depicted in Figure 1.

**Table 2:** Demographics of the Healthy Volunteers (*n* = 89)

Demographic	
Age (years)*	44.8 ± 18.0
Age ≥70 years, *n* (%)	9 (10)
Sex (male) *n* (%)	45 (50)
Height (cm)*	170 ± 10
Weight (kg)*	75.9 ± 15.0
Body mass index, kgm^−2^	26 ± 4
Body surface area (m^2^)*	1.87 ± 0.20

*Mean ± SD.

**Table 3:** Comparison of Heart Rate and LV Parameters at 1.5T and 3.0T

	1.5T	3.0T	*P*‐value*
Heart rate (bpm)	65.6 ± 11	64.2 ± 11	0.131
LVEF (%)	63.6 ± 5	63.4 ± 5	0.508
LVEDV index (mL/m^2^)	69.8 ± 11	70.7 ± 11	0.205
LVESV index (mL/m^2^)	25.6 ± 6	26.2 ± 6	0.106
LV mass index (g/m^2^)	40.3 ± 10	41.1 ± 10	0.051

LVEF: Left ventricle ejection fraction; LVESV: Left ventricle end‐diastolic volume; LVESV: Left ventricle end‐systolic volume.

*Measured using Student's *t*‐test.

### Artifact Analysis and Interobserver Analysis

Overall image quality was high for circumferential (94%) and longitudinal strain (92%) at 1.5T (Table 4). Overall quality was adequate at 3.0T for both circumferential (54%) and longitudinal strain (57%). 7% of circumferential and 9% of longitudinal strain data could not be analyzed due to poor quality at 3.0T, while all images were of diagnostic quality at 1.5T. A higher proportion of images had artifact at 3.0T when compared with 1.5T, as can be seen in Table 4.

**Table 4:** Image Quality and Artifact Scoring

DENSE sequences	1.5T		3.0T	
Image quality	E_cc_ (*n* = 81)	E_ll_ (*n* = 34)	E_cc_ (*n* = 88)	E_ll_ (*n* = 86)
High quality^a^	76 (94%)	31 (92%)	34 (39%)	28 (33%)
Adequate quality^b^	5 (6%)	3 (8%)	48 (54%)	49 (57%)
Nondiagnostic^c^	0 (0%)	0 (0%)	6 (7%)	9 (10%)
Number of images with motion artifact	2 (2%)	2 (6%)	40 (45%)	43 (50%)
Number of images with field effect artifact	1 (1%)	0 (0%)	11 (13%)	13 (15%)

a High quality: Well defined endo‐ and epicardial borders at end‐systole. No ghosting due to patient breathing. No flow artifact.

b Adequate quality: One or more of the following were present: Slight blurring of endo‐ and epicardial borders at end‐systole. Slight artifact due to flow within blood pool but not affecting myocardium. Slight ghosting due to patient breathing.

c Nondiagnostic: One or more of the following are present: Loss of endo‐ and epicardial borders at end‐systole; ghosting due to respiration.

Interoperator repeatability analysis was performed on the strain analysis, and the intraclass correlation coefficient was above 0.85 for all parameters (Table 5).

**Table 5:** Reproducibility of DENSE Analysis

Variable	Mean bias ± SD (%)	95% Limits of agreement	*P*‐value	CoV	ICC
E_cc_ 1.5T	0.2 ± 1.2	−2.1 to 2.5	0.45	4.3	0.93
E_ll_ 1.5T	0.5 ± 2.1	−3.7 to 4.7	0.33	12.5	0.86
E_cc_ 3.0T	0.4 ± 1.4	−2.4 to 3.1	0.27	5.6	0.93
E_ll_ 3.0T	0.6 ± 2.3	−3.9 to 5.0	0.29	13.7	0.89

E_cc_: circumferential strain; E_ll_: longitudinal strain; ICC: intra‐class correlation co‐efficient. The inter observer variability analysis. A sample size of 20 images was taken per variable. The intra‐class correlation coefficient is above 0.85 for all of them.

### Sex Differences in LV Strain at 1.5 and 3.0T

Global circumferential and longitudinal strains were greater in magnitude in women than in men at both field strengths (Table 6). Regional differences in strain were also observed. Longitudinal strain values were greater in the apico‐septal, basal‐lateral, and mid‐lateral segments and circumferential strain in the inferior, infero‐lateral, and antero‐lateral LV segments.

**Table 6:** Circumferential and Longitudinal Strain at 1.5 and 3.0T in Males and Females

	1.5T			3.0T		
Parameter	Male	Female	*P*‐value	Male	Female	p‐value
E_cc_*	(*n* = 39)	(*n* = 42)		(*n* = 41)	(*n* = 41)	
*Global* (%)	*‐18.7 ± 2.2*	*‐20.1 ± 2.7*	*0.014*	*‐17.9 ± 1.8*	*‐19.1 ± 3.1*	*0.047*
Anterior (%)	−20.3 ± 3.5	−21.6 ± 4.2	0.153	−18.7 ± 3.7	−21.0 ± 3.9	0.008
Antero‐septal (%)	−18.2 ± 3.7	−17.9 ± 3.3	0.667	−17.0 ± 2.9	−17.9 ± 3.7	0.245
Infero‐septal (%)	−16.0 ± 3.6	−17.4 ± 3.0	0.055	−15.4 ± 3.0	−15.9 ± 4.1	0.511
Inferior (%)	−18.5 ± 3.3	−20.8 ± 3.6	0.003	−19.3 ± 3.4	−20.3 ± 4.3	0.246
Infero‐lateral (%)	−21.1 ± 2.9	−22.7 ± 3.7	0.030	−21.0 ± 3.1	−20.8 ± 4.7	0.851
Antero‐lateral (%)	−20.9 ± 3.2	−22.3 ± 3.2	0.047	−19.2 ± 3.6	−21.4 ± 4.2	0.012
E_ll_*	(*n* = 14)	(*n* = 20)		(*n* = 39)	(*n* = 38)	
*Global* (%)	*‐10.2 ± 3.4*	*‐13.7 ± 2.4*	*0.001*	*‐10.7 ± 2.4*	*‐11.9 ± 2.7*	*0.052*
Basal‐septal (%)	−8.2 ± 5.1	−10.7 ± 4.8	0.151	−9.0 ± 4.2	−10.3 ± 4.3	0.163
Mid‐septal (%)	−12.4 ± 2.8	−13.2 ± 4.4	0.550	−12.2 ± 3.6	−11.9 ± 4.2	0.803
Apico‐septal (%)	−12.7 ± 4.5	−16.4 ± 4.8	0.03	−15.1 ± 4.9	−16.7 ± 4.0	0.115
Basal‐lateral (%)	−12.9 ± 3.1	−15.8 ± 4.0	0.027	−12.8 ± 4.4	−15.5 ± 4.7	0.01
Mid‐lateral (%)	−12.7 ± 4.5	−15.9 ± 3.3	0.02	−12.5 ± 4.3	−12.8 ± 4.4	0.747
Apico‐lateral (%)	−13.3 ± 3.9	−15.0 ± 3.4	0.176	−13.6 ± 4.3	−13.5 ± 4.6	0.933

Mean (± SD); E_cc_: Circumferential strain; E_ll_: Longitudinal strain.

### Age Differences in LV Strain at 1.5 And 3.0T

Due to the sex difference in strain we carried out a regression model with multiple variables (age and sex) (Tables 6, 7). At 1.5T, longitudinal and circumferential strain were not associated with age after accounting for sex. At 3.0T, there was a statistically significant relationship between both longitudinal and circumferential strain and age (*P* < 0.05). We have also presented global and segmental strain according to three age tertiles (Table 8).

**Table 7:** Significance of Relationship of Strain With Age and Sex at 1.5T and 3.0T

	1.5T		3.0T	
	E_cc_	E_ll_	E_cc_	E_ll_
*P* value for regression	0.046	0.002	0.008	0.004
*P* value for age	0.733	0.178	0.017	0.007
*P* value for sex	0.015	0.001	0.029	0.033

E_cc_: circumferential strain; E_ll_: longitudinal strain.

**Table 8:** Magnitudes of Strain Per Age Tertile

	1.5T			3.0T		
Parameter	1^st^ tertile (<35 y)	2^nd^ tertile (35–55 y)	3^rd^ tertile (>55y)	1^st^ tertile (<35 y)	2^nd^ tertile (35–55 y)	3^rd^ tertile (>55y)
Ecc*	*n* = 31	*n* = 27	*n* = 26	*n* = 31	*n* = 30	*n* = 26
*Global* (%)	−19.4	−19.4	−19.3	−18.7	−18.6	−18.4
Anterior (%)	−18.1	−17.9	−17.9	−17.6	−17.5	−17.5
Antero‐septal (%)	−21.0	−20.9	−20.8	−19.7	−19.7	−19.8
Infero‐septal (%)	−21.5	−21.6	−20.6	−20.2	−20.4	−20.1
Inferior (%)	−22.0	−21.9	−21.8	−21.1	−21.0	−20.7
Infero‐lateral (%)	−19.7	−19.4	−19.5	−20.1	−20.0	−19.5
Antero‐lateral (%)	−16.7	−16.6	−16.7	−15.7	−15.7	−15.4
Ell*	*n* = 12	*n* = 11	*n* = 11	*n* = 30	*n* = 30	*n* = 22
*Global* (%)	−11.4	−12.3	−12.2	−12.3	−11.3	−11.0
Basal‐septal (%)	−9.8	−9.7	−9.5	−9.8	−9.6	−9.3
Mid‐septal (%)	−12.2	−12.9	−12.8	−12.8	−12.0	−11.6
Apico‐septal (%)	−15.7	−14.9	−14.7	−15.1	−15.7	−15.6
Basal‐lateral (%)	−14.0	−14.6	−14.7	−14.6	−14.1	−14.0
Mid‐lateral (%)	−12.8	−14.6	−14.5	−14.3	−13.0	−12.4
Apico‐lateral (%)	−13.7	−14.3	−14.5	−14.2	−13.4	−13.6

E_cc_: circumferential strain; E_ll_: longitudinal strain.

### Myocardial Strain: Variations With Field Strength

Global and regional strain values differed slightly between 1.5T and 3.0T (Fig. 1). These differences mainly related to circumferential strain values in the anterior, infero‐lateral, and antero‐lateral LV segments, where strain is greater at 1.5T. Global longitudinal strain and regional longitudinal strain in the mid‐septal and medio‐lateral segments were higher at 1.5T compared with 3.0T.

## Discussion

We described myocardial strains at different MRI field strengths in 89 healthy adults across a broad age range, including 10% of elderly individuals ≥70 years. Circumferential and longitudinal strain values revealed by 2D‐DENSE had low interobserver variability, implying good reliability. We observed that longitudinal and circumferential strains varied in a regional distribution, with higher strain values in the anterior and lateral LV territories. Strains were higher in females than in males. After adjusting for the association with sex, strain was only associated with age when assessed at 3.0T. We also observed small differences in the magnitude of longitudinal and circumferential strains at different field strengths, with the magnitude of strain being slightly higher at 1.5T than 3.0T.

We observed that females had higher magnitudes of peak strain when compared with males. These differences were statistically significant for circumferential strain at both 1.5T and 3.0T and for longitudinal strain at 1.5T. Longitudinal strain at 3.0T was numerically higher for females, with a *P* value approaching statistical significance. These results are similar to what has been published in previous studies by Lawton et al^16^ using tagging, and Taylor et al^17^ using feature‐tracking. On the other hand, other authors, including Augustine et al,^18^ using feature‐tracking, Neizel et al^19^ using SENC, and Kuznetsova et al^20^ using echocardiography found that women had similar circumferential and longitudinal strain to men. DENSE,^1^ tagging,^8^ phase contrast MR,^10^ and feature tracking^21^ use different techniques to analyze strain, which could account for some of the differences in strain values observed using these different methods.^22^

We used a multivariate regression model to account for sex differences to assess whether there was an age‐related change in strain. Interestingly, there was a statistically significant relationship between age and strain at 3.0T but not at 1.5T. On reviewing the literature, it seems that there is no clear consensus on the relationship between age and strain. Oxenham et al^23^ and Neizel et al^19^ (Supplementary Table 1) did not observe any association between age and strain when assessed using tagged MR. Taylor et al^17^ described a positive association between circumferential strain but not longitudinal strain with age in individuals over the age of 50 years. Kuznetsova et al^20^ using echocardiography in 236 healthy subjects found that strain was inversely associated with age, consistent with the results at 3.0T in our study.

We observed that myocardial strain measured using DENSE was higher at 1.5T compared with 3.0T. Looking to the literature, Schuster et al.^24^ and Wehner et al.^25^ did not observe any differences in strain between field strengths; however, the sample size in both studies was small.

The 3.0T scanner used in this study had a 70 cm bore, while the 1.5T had a 60 cm bore. During scan acquisition, we found that it was more difficult to obtain a high level of magnetic field homogeneity in a wide bore scanner and hence artifacts were more common. The breath‐hold duration with this DENSE application may have lasted up to 20 seconds, depending on the heart rate, which may have resulted in motion artifacts due to respiration. Markl et al^26^ (*n* = 16) compared image quality using tagging at both field strengths and reported an increase in off‐resonance artifact at 3.0T, without an effect on overall image quality. Kramer et al^27^ (*n* = 14) and Valeti et al^28^ (*n* = 13) reported better image quality with tagging at 3.0T.

Another potentially relevant factor is that the timing parameters, eg, TR, of the DENSE pulse sequence were slightly different between the two field strengths. Since sampling of strain during the cardiac cycle occurred at slightly different timepoints, it is possible that there may have been a small disparity in what was perceived to be the “peak” strain at 1.5T and 3.0T. Importantly, the magnitude of the differences in strain between the field strengths was small and unlikely to be clinically significant; however, this result means that we need to interpret the observed relationship between age and strain at 3.0T with caution.

A summary of studies looking at strain assessment in healthy volunteers is provided in Supplementary Table 1. Compared with these studies, the distinct features of our study are 1) healthy adults with no history of cardiovascular disease or treatment were recruited; 2) further verification and exclusion of subclinical cardiac disease by the requirement of a normal ECG and exclusion of patients with incidental myocardial fibrosis or scar; 3) a reasonably large cohort of adults equally balanced between male:female and representative of a broad range of ages; and 4) performance of MR at 1.5T and 3.0T within a short period of time (average 1.4 days).

Reproducibility with EPI DENSE in our study seems to be higher than with feature Tracking and Tagging. For feature tracking at 1.5T,^16^ an interobserver coefficient of variation between 5.48% and 17.3% was reported for longitudinal strain, and between 4.9% and 13.3% for circumferential strain. For feature tracking at 3.0T, an interobserver coefficient of variation between 16.3% and 26.4% for longitudinal strain and between 9.9% and 20.3% for circumferential strain^17^, ^18^, ^29^, ^30^ was reported. Reproducibility with DENSE is higher than with Tagging.^30^ Compared to spiral cine DENSE,^25^ the reproducibility in our study was similar for circumferential strain and lower for longitudinal strain.

Strain measurements with MRI provide clinically useful information linked with myocardial viability^31^, ^32^ and predictive of longer‐term prognosis.^33^, ^34^, ^35^ Strain derived from DENSE provides higher spatial density of displacement and a higher temporal resolution compared to other MRI sequences.^24^ On the other hand, MRI has a lower temporal resolution compare to echocardiography, but MR permits tissue characterization. DENSE MRI has potential for wider adoption in clinical practice, and our study provides further information that adds to what is known about myocardial contractility revealed by DENSE MRI, including field‐ and sex‐specific differences.

Strain imaging is emerging as an alternative to LVEF and wall motion for the assessment of myocardial function. In the American Society of Echocardiography and the European Association of Cardiovascular Imaging guidelines, serial longitudinal strain assessment is recommended for assessment of patients treated with cytotoxic chemotherapy.^37^ Strain‐echocardiography has emerging utility for the discrimination of hypertrophic cardiomyopathy from hypertensive LV hypertrophy.^38^ DENSE MRI adds diagnostically over echocardiography because of the potential to characterize tissue pathology and register with contractility in the same scan. Accordingly, DENSE MRI has future potential in clinical practice. There have been further methods development using DENSE since the 2D EPI‐DENSE, including increasing signal‐to‐noise ratio by spiral *k*‐space acquisition,^39^ increasing phase signal‐to‐noise ratio by balanced encoding^13^ artifact suppression^14^ to improve 2D DENSE, as well as work on 3D‐DENSE whole heart acquisition.^40^

Fewer participants had longitudinal DENSE data acquired at 1.5T because 1.5T scans were performed on an MRI scanner in a busy cardiac regional center, whereas the 3.0T scans were obtained on a research‐dedicated scanner that had greater flexibility for the duration of the scan. Our study was exploratory. The results are hypothesis generating and further research is warranted.

In conclusion, we described myocardial strains at different MR field strengths in reasonably large sample of healthy adults across a broad age range with cine DENSE MR. We observed that longitudinal and circumferential strains varied in a regional distribution, with higher strain values in the anterior and lateral LV territories. Strains were higher in females than in males. Accounting for the between‐sex difference an association between age and strain was only apparent at 3.0T. We also observed small differences in the magnitude of longitudinal and circumferential strains at different field strengths, with the magnitude of strain being slightly higher at 1.5T than 3.0T.

## Potential Conflict of Interest

The University of Glasgow holds a research agreement with Siemens Healthcare, who provided the DENSE work‐in‐progress CMR sequence and data analysis software.

## Author contributions

CB, CM, DC made substantial contributions to conception and design; DC, CM, KM, RW, VO made substantial contributions to acquisition of data; KM, GC, CM, DSC, SR, JM made substantial contributions to the analysis and interpretation of data; KM, GC, CM, XZ, CB drafted the article; KM, GC, CB, XZ, CM, RW, VO, JM were involved in revising it critically for important intellectual content. All authors gave final approval of the version to be submitted and any revised version.

## Supporting information

Supporting Information
