# Supplementary material for: Myocardial strain in healthy adults across a broad age range as revealed by cardiac magnetic resonance imaging at 1.5 and 3.0T: Associations of myocardial strain with myocardial region, age, and sex
Source: J Magn Reson Imaging. 2016 Apr 22;44(5):1197–205. doi: 10.1002/jmri.25280 (PMC5082565; doi:10.1002/jmri.25280)
Supplement: Supplementary file 1 — Supporting Information [file JMRI-44-1197-s001.docx]

| Author | Strain method used | Field strength | Sample size | Age (y ± SD) | Sex (% male) | Peak Ecc | Peak Ell |
| --- | --- | --- | --- | --- | --- | --- | --- |
| Augustine, 2013 (53) | feature-tracking | 1.5T | 145 | 30 ± 8 | 37 | -0.21 ± 0.03 | -0.19 ± 0.003 |
| Clark, 1991 (54) | tagging | 1.5T | 15 | 23 ± 34 | 33 | not provided | not provided |
| Cupps, 2010 (55) | tagging | 1.5T | 50 | 33 ± 11 | not provided | not provided | not provided |
| Feng, 2009 (56) | DENSE | 3T | 12 | 35 ± 11 | 58 | -18.7 |  |
| Hamdan, 2009 (57) | SENC | 3T | 16 | 34 ± 2 | 88 | not provided | not provided |
| Harrild, 2011 (58) | tagging, feature-tracking | 1.5T | 13 | 32 ± 15 | 62 | -23 ± 2 ; -21 ± 4 | not provided |
| Kar, 2014 (59) | DENSE, tagging | 1.5T | 12 | not provided | not provided | -16.8 ; -16 | not provided |
| Korosoglou 2008 (60) | SENC, tagging | 3T | 12 | 44 ± 9 | 75 | -21.7 ± 2.7 ; -22.9 ± 3.1 | not provided |
| Kramer, 1994 (61) | tagging | 1.5T | 10 | 27 ( SD not provided) | not provided | not provided | not provided |
| Lawton, 2011 (29) | tagging | 1.5T | 60 | 33 ± 11 | 46 | Male -19 ± 0.02 ; Female-21 ± 0.02 | Male -14 ± 0.03 ; Female -16.± 0.02 |
| Moore, 2000 (62) | tagging | 1.5T | 31 | 37 ± 11 | 52 | not provided | not provided |
| Neizel, 2009 (12) | SENC, tagging | 1.5T | 75 | 44 ± 12 | 53 | not provided | not provided |
| Oxenham, 2003 (26)) | tagging | 1.5T | 31 | 46 | not provided | not provided | not provided |
| Palmon, 1994 (63) | tagging | 1.5T | 21 | 29 | 48 | not provided | not provided |
| Rogers, 1991 (64) | tagging | 0.38T | 19 | 56 ± 17 | 74 | not provided | not provided |
| Schuster, 2013 (16)00 | feature-tracking | 1.5T, 3.0T | 10 | 31y at 1.5T, 41y at 3.0T | 50 | 1.5T: -19.7 ± 7.7  3.0T: -18.7 ± 9.5 | \|  \| \| --- \|   1.5T: - 18.8 ± 10.6  3.0T: - 20.1 ± 10.3 |
| Taylor, 2015 (27)) | feature-tracking | 1.5T | 100 | 45 ± 14 | 50 | -26.1 ± 3.8 | -21.3 ± 4.8 |
| Wehner, 2015 (34) | DENSE, tagging, | 1.5T, 3.0T | 10 | 29 ± 4 | 60 | not provided | 1.5T : -12.3 ± 1.8 ; -14.3 ± 1.7  3T : -14.7 ± 1.4 ; -13.0 ± 1.2 |
| Wu, 2014 (65) | tagging | 1.5T | 10 | 37 ± 11 | 90 | 16.5 ± 1.6 | not provided |
| Young, 2012 (66) | DENSE, tagging | 1.5T | 19 | range 23-49 | 19 | not provided | not provided |

## Supplementary Table 1: Publications describing myocardial strain in healthy volunteers with a sample size ≥ 10. * Ecc- circumferential strain, Ell- longitudinal strain.

### Supplementary Results: Missing data and study participant characteristics

88 volunteers attended for a 3.0T CMR. The age (mean ± standard deviation; range) of the participants was 44.6 ± 18, 18 to 87 years old, and 43 (49%) were male.

89 volunteers attended for a 1.5T CMR. The age (mean ± standard deviation; range) of the participants was 44.8 ± 18 and 44 (49%) were male.

77 volunteers had longitudinal strain analysable at 3.0T; the age (mean ± standard deviation; range) of the participants was 44.5 ± 18, 18 to 87 years old, and 39 (51%) were male.

34 volunteers had longitudinal strain analysable at 1.5T, due to time pressures. The age (mean ± standard deviation; range) of the participants was 47.1 ± 20; 18 to 87 years and 14 (41%) were male.

82 volunteers had circumferential strain analysable at 3.0T; the age (mean ± standard deviation; range) of the participants was 45.0 ± 18, 18 to 87 years old, and 41 (50%) were male.

81 volunteers had circumferential strain analysable at 1.5T, due to time pressures. The age (mean ± standard deviation; range) of the participants was 44.9 ± 18; 18 to 87 years and 39 (48%) were male.

### Supplementary References:

1. Augustine D, Lewandowski AJ, Lazdam M, Rai A, Francis J, Myerson S, et al. Global and regional left ventricular myocardial deformation measures by magnetic resonance feature tracking in healthy volunteers: comparison with tagging and relevance of gender. J Cardiovasc Magn Reson. 2013 Jan 18;15(1):8.

2. Clark NR, Reichek N, Bergey P, Hoffman EA, Brownson D, Palmon L, et al. Circumferential myocardial shortening in the normal human left ventricle. Assessment by magnetic resonance imaging using spatial modulation of magnetization. Circulation. 1991 Jul 1;84(1):67–74.

3. Cupps BP, Taggar AK, Reynolds LM, Lawton JS, Pasque MK. Regional Myocardial Contractile Function: Multiparametric Strain Mapping. Interact Cardiovasc Thorac Surg. 2010 Jun;10(6):953–7.

4. Feng L, Donnino R, Babb J, Axel L, Kim D. Numerical and In Vivo Validation of Fast Cine DENSE MRI for Quantification of Regional Cardiac Function. Magn Reson Med Off J Soc Magn Reson Med Soc Magn Reson Med. 2009 Sep;62(3):682–90.

5. Hamdan A, Thouet T, Kelle S, Wellnhofer E, Paetsch I, Gebker R, et al. Strain-encoded MRI to evaluate normal left ventricular function and timing of contraction at 3.0 Tesla. J Magn Reson Imaging JMRI. 2009 Apr;29(4):799–808.

6. Harrild DM, Han Y, Geva T, Zhou J, Marcus E, Powell AJ. Comparison of cardiac MRI tissue tracking and myocardial tagging for assessment of regional ventricular strain. Int J Cardiovasc Imaging. 2012 Dec;28(8):2009–18.

7. Kar J, Knutsen AK, Cupps BP, Zhong X, Pasque MK. Three-dimensional regional strain computation method with displacement encoding with stimulated echoes (DENSE) in non-ischemic, non-valvular dilated cardiomyopathy patients and healthy subjects validated by tagged MRI. J Magn Reson Imaging. 2015 Feb 1;41(2):386–96.

8. Korosoglou G, Youssef AA, Bilchick KC, Ibrahim E-S, Lardo AC, Lai S, et al. Real-time fast strain-encoded magnetic resonance imaging to evaluate regional myocardial function at 3.0 Tesla: comparison to conventional tagging. J Magn Reson Imaging JMRI. 2008 May;27(5):1012–8.

9. Kramer CM, Reichek N, Ferrari VA, Theobald T, Dawson J, Axel L. Regional heterogeneity of function in hypertrophic cardiomyopathy. Circulation. 1994 Jul 1;90(1):186–94.

10. Lawton JS, Cupps BP, Knutsen AK, Ma N, Brady BD, Reynolds LM, et al. Magnetic resonance imaging detects significant sex differences in human myocardial strain. Biomed Eng OnLine. 2011 Aug 22;10(1):76.

11. Moore CC, Lugo-Olivieri CH, McVeigh ER, Zerhouni EA. Three-dimensional Systolic Strain Patterns in the Normal Human Left Ventricle: Characterization with Tagged MR Imaging. Radiology. 2000 Feb;214(2):453–66.

12. Neizel M, Lossnitzer D, Korosoglou G, Schäufele T, Lewien A, Steen H, et al. Strain-encoded (SENC) magnetic resonance imaging to evaluate regional heterogeneity of myocardial strain in healthy volunteers: Comparison with conventional tagging. J Magn Reson Imaging JMRI. 2009 Jan;29(1):99–105.

13. Oxenham HC, Young AA, Cowan BR, Gentles TL, Occleshaw CJ, Fonseca CG, et al. Age-related changes in myocardial relaxation using three-dimensional tagged magnetic resonance imaging. J Cardiovasc Magn Reson Off J Soc Cardiovasc Magn Reson. 2003 Jul;5(3):421–30.

14. Palmon LC, Reichek N, Yeon SB, Clark NR, Brownson D, Hoffman E, et al. Intramural myocardial shortening in hypertensive left ventricular hypertrophy with normal pump function. Circulation. 1994;89(1):122–31.

15. Rogers WJ, Shapiro EP, Weiss JL, Buchalter MB, Rademakers FE, Weisfeldt ML, et al. Quantification of and correction for left ventricular systolic long-axis shortening by magnetic resonance tissue tagging and slice isolation. Circulation. 1991;84(2):721–31.

16. Schuster A, Morton G, Hussain ST, Jogiya R, Kutty S, Asrress KN, et al. The intra-observer reproducibility of cardiovascular magnetic resonance myocardial feature tracking strain assessment is independent of field strength. Eur J Radiol. 2013 Feb;82(2):296–301.

17. Taylor RJ, Moody WE, Umar F, Edwards NC, Taylor TJ, Stegemann B, et al. Myocardial strain measurement with feature-tracking cardiovascular magnetic resonance: normal values. Eur Heart J Cardiovasc Imaging. 2015 Feb 23;

18. Wehner GJ, Suever JD, Haggerty CM, Jing L, Powell DK, Hamlet SM, et al. Validation of in vivo 2D displacements from spiral cine DENSE at 3T. J Cardiovasc Magn Reson. 2015 Jan 30;17(1):5.

19. Wu L, Germans T, Güçlü A, Heymans MW, Allaart CP, Rossum AC van. Feature tracking compared with tissue tagging measurements of segmental strain by cardiovascular magnetic resonance. J Cardiovasc Magn Reson. 2014 Jan 22;16(1):10.

20. Young AA, Axel L, Dougherty L, Bogen DK, Parenteau CS. Validation of tagging with MR imaging to estimate material deformation. Radiology. 1993 Jul;188(1):101–8.
